# Island Life: Use of Activity Budgets and Visibility to Evaluate a Multi-Species Within-Zoo Exhibit Move

**DOI:** 10.3390/ani12162123

**Published:** 2022-08-19

**Authors:** Katherine Finch, James O. Waterman, Veronica B. Cowl, Ashleigh Marshall, Lydia Underwood, Leah J. Williams, Nick Davis, Lisa Holmes

**Affiliations:** 1 North of England Zoological Society (Chester Zoo), Cedar House, Caughall Road, Chester CH2 1LH, UK; 2 Faculty of Biology, Medicine and Health, University of Manchester, Oxford Road, Manchester M13 9PL, UK; 3 Department of Natural Sciences, Manchester Metropolitan University, John Dalton Building, Chester Street, Manchester M1 5GD, UK

**Keywords:** zoo, animal management, evidence based, enclosure design, animal welfare, natural behaviours, animal behaviour

## Abstract

**Simple Summary:**

Modern zoos aim to provide the best facilities possible for their animals, staff and visitors. Here, we present findings from a study focusing on the behaviour and visibility of four species pre- and post-translocation to a new environment, *Islands,* within Chester Zoo, UK: the Sumatran orangutan (*Pongo abelii*), crested macaque (*Macaca nigra*), Malayan tapir (*Tapirus indicus*) and the Malayan sun bear (*Helarctos malayanus*). We used full activity budgets to demonstrate that the move to new, custom-built facilities influenced the behaviour of all four species. Following relocation, both non-human primate species were found to spend more time interacting socially with group members and abnormal behaviours remained low for all four species. Malayan tapirs and crested macaques chose to spend more time in areas out of public view post-move, whilst Malayan sun bears were more visible to visitors in their new environment. We demonstrate the value of giving animals choice and control over how they interact with their surroundings, the importance in investment in behavioural monitoring throughout translocation events and add to the knowledge-base of this understudied area.

**Abstract:**

Modern zoos strive to construct habitats which both enable and encourage animals to engage in species-specific behaviour, without compromising their visibility to visitors. Here, we present the findings of a within-zoo move to a custom-built exhibit (*Islands* at Chester Zoo, UK) with respect to the behaviour of four mammal species; the Sumatran orangutan (*Pongo abelii*), crested macaque (*Macaca nigra*), Malayan tapir (*Tapirus indicus*) and the Malayan sun bear (*Helarctos malayanus*). We used full activity budgets along with Compositional Data Analysis (CoDA) to gain insight into how the move to a more naturalistic exhibit influenced behaviour. Engagement in abnormal behaviour remained low during the study period for all four species, suggesting no adverse responses to the change in environment. Following the move, both the non-human primate species spent more time engaged in positive social interactions with conspecifics, highlighting the importance of social support during enclosure moves. Time spent visible to the public was largely unaffected by the enclosure move for the Sumatran orangutan, whilst the movement to a new environment increased visibility for the Malayan sun bear and decreased visibility for the crested macaque and Malayan tapir. We demonstrate the value of monitoring behaviour throughout the translocation of zoo-housed species and outline the positive behavioral impacts of providing individuals with naturalistic, species-appropriate environments.

## 1. Introduction

Successful exhibit design can enable modern zoos to achieve many of their strategic- and conservation-related objectives by providing species-appropriate habitats for their animals and learning opportunities for visitors [1]. As naturalistic habitat design becomes more common in new animal facilities, it is important to consider a species’ natural history in the planning process [2]. Providing an environment with adequate space and complexity requires an in-depth knowledge of an animals’ typical home-range and behaviour [3], which can often only be obtained from experienced practitioners [4]. A failure to acknowledge this key information during the exhibit design process can have a detrimental effect on the animals that subsequently inhabit that space [5]. Inadequate facilities are associated with abnormal repetitive behaviours [6], poor body condition [7], low reproductive success [8] and in some cases death [9]. Additionally, poor facility design may not encompass the needs of animal keepers to implement targeted, species-appropriate enrichment schedules or management routines, potentially further compromising welfare [10].

Despite the potentially negative effects of sub-optimal housing, research on the effect of a move to a novel, more naturalistic environment remains limited [11]. Environments with a naturalistic design have been shown to increase visitor dwell time [12], and visitors have ranked animal welfare higher in these exhibits than in more barren areas [4]. Following transfer to new naturalistic exhibits, several effects have been observed: individual activity levels increase [13,14,15], as does foraging, locomotion [16] and species-typical behaviour [17]. However, there is a clear taxonomic bias within this research, with non-human primates (hereafter primates) being the most studied group [11]. As such, research on a broader range of species is required.

Animal welfare should be of paramount importance to any modern zoological organisation [18,19], and engagement in species-typical behaviour can be a useful tool in the evaluation of the welfare state [20]. The expression of species-typical behaviour is associated with positive contributions to conservation breeding programs [21], suitability for conservation reintroduction attempts [21] and a reduction in the incidence of health issues [22]. Thus, the documentation of these behaviours forms an essential component of any welfare monitoring process. Accurate evaluation of behaviour post-exhibit move using robust and consistent methodology will enable the application of an evidence-based approach to species management and exhibit design [23,24,25]. Additionally, because behavioural changes in response to enclosure moves may not be immediately apparent [26] or may be an initial response to a novel situation [27,28], it is important to account for a habituation period to management or environment modifications.

This study evaluates the effects of an enclosure move on the behaviour and visibility of four species at Chester Zoo, UK. In 2015, Chester Zoo opened a new development named *Islands*. *Islands* aimed to provide a naturalistic and immersive visitor experience by replicating the island habitats of South-East Asia. Many species were transferred from the core zoo to new, custom-built enclosures within *Islands* throughout the multi-year development. Four of the flagship species for this expansion were the Sumatran orangutan (*Pongo abelii*), crested macaque (*Macaca nigra*), Malayan tapir (*Tapirus indicus*) and the Malayan sun bear (*Helarctos malayanus*); all species threatened with extinction in their native habitat and popular with the visiting public. Here, we aimed to conduct a multi-species evaluation of a move to a new, more natural environment within the *Islands* expansion at Chester Zoo, UK. By completing this study, we aim to show the benefit of post-translocation monitoring within a zoo, address the literature gap on the behavioural influence of exhibit moves and provide an evidence base that can be shared with internal and external stakeholders. We made the following predictions: movement to a new custom-built facility would influence the activity budget of all four species (P.1) and the visibility of the species to visitors would be affected by the change in environment (P.2). Furthermore, we predicted that there would be a period of habituation to the new environment by the study subjects (P.3). 

## 2. Materials and Methods

### 2.1. Study Individuals

Twenty four individuals from 4 species were studied over a total of 33 months (January 2015 to September 2017), all housed at Chester Zoo, UK. Species were moved into their new enclosures on different dates, but pre- and post-move data were collected for all during this period (see Table 1 for the details of the study subjects and data collection periods and Appendix A for pre- and post-move enclosure designs). Husbandry routines remained similar between the pre- and post-move conditions.

### 2.2. Exhibit Information

All areas and volumes provided are approximations, based on exhibit drawings (Appendix B).

#### 2.2.1. Sumatran Orangutan Exhibit Information

**Figure A1:**
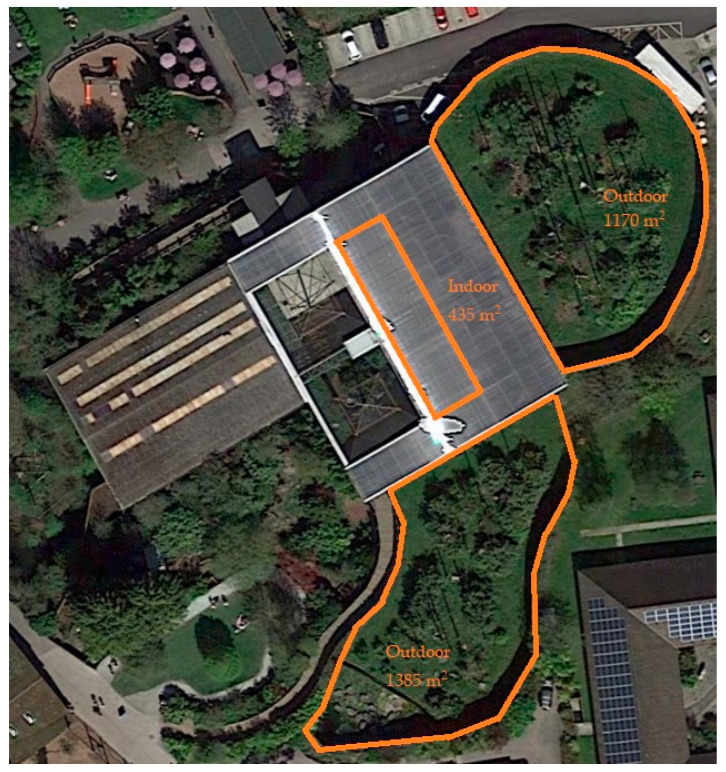
Enclosure plan for Sumatran orangutans (*Pongo abelii*) in the *Realm of the Red Ape* exhibit in which individuals were housed during the pre-move data collection period. Orange lines signify approximate areas accessed by individual animals with dimensions of total available space. Copyright: Google Earth.

**Figure A2:**
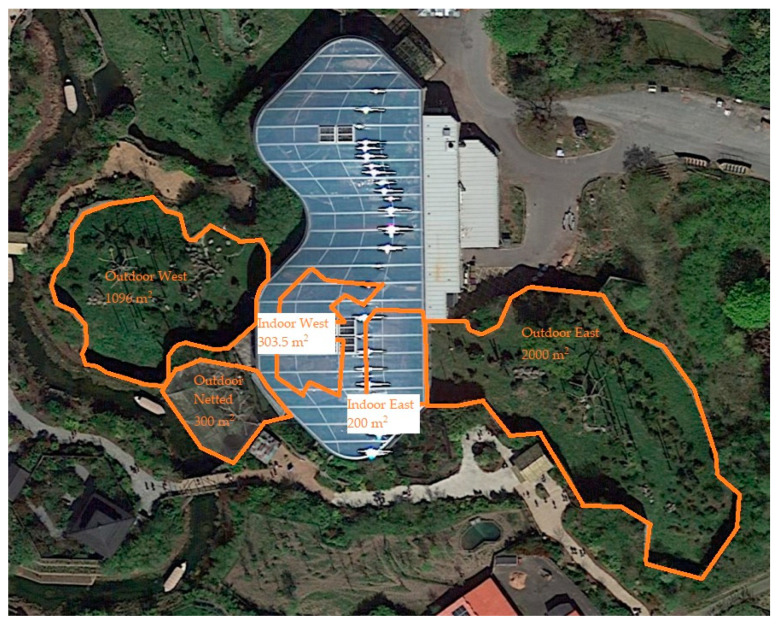
Enclosure plan for Sumatran orangutan (*Pongo abelii*) in *Monsoon Forest*, within the *Islands* expansion. Orange lines signify approximate areas accessed by individual animals with dimensions of total available space. During data collection periods, individuals only had access to Outdoor West, Indoor West and Outdoor Netted areas. Copyright: Google Earth.

During the pre-move condition, this species was held in *Realm of the Red Ape*—a custom-built orangutan facility constructed in 2007 (Appendix B, Figure A1). Individuals had access to three indoor areas, each with an area of 145 m^2^ and a cubic volume of 1435 m^3^. Indoor areas focused on providing vertical, three-dimensional space to facilitate arboreal activity. Additionally, individuals had access to two open-air outdoor exhibits (1385 m^2^ and 1170 m^2^). Species-appropriate features included mesh ropes, hammocks and wooden poles to encourage arboreal locomotion. During the post-move condition, this species was housed within *Monsoon Forest*, an area including a biodome within *Islands*. Here, individuals had access to two indoor enclosures (totalling 303.5 m^2^ area, 1816 m^3^ volume), one open air outdoor enclosure (1096 m^2^ area) and one netted outdoor exhibit (300 m^2^ area, 2450 m^3^ volume) (Appendix B, Figure A2). A further indoor area (200 m^2^ area, 910 m^3^ volume) and one further open-air exhibit (2000 m^2^ area) were made available to this species after the post-move data collection ended. Species-specific enclosure features included fiber-glass sway poles, larger artificial tree climbing structures, custom mesh hammocks to increase nesting and resting opportunities, varying height levels throughout indoor environments and heavy planting [29,30,31].

#### 2.2.2. Crested Macaque Exhibit Information

**Figure A3:**
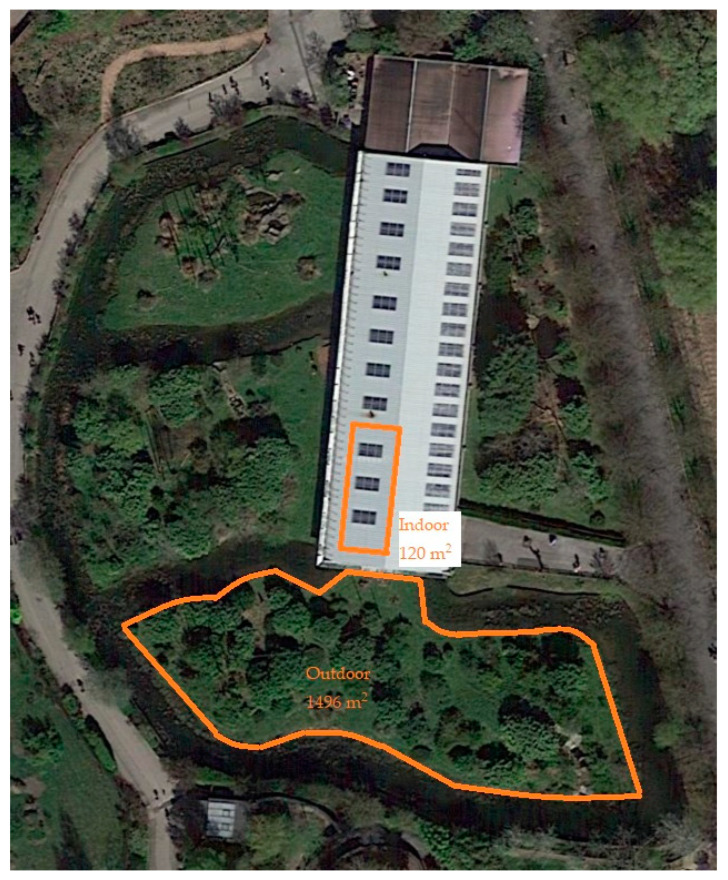
Enclosure plan for Sulawesi crested macaques (*Macaca nigra*) in the *Monkey House* exhibit. Orange lines signify approximate areas accessed by individual animals with dimensions of total available space. Copyright: Google Earth.

**Figure A4:**
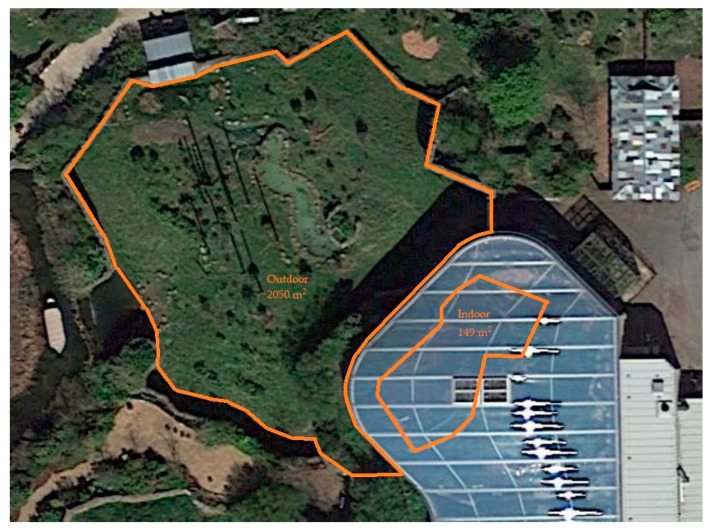
Enclosure plan for Sulawesi crested macaques (*Macaca nigra*) in *Monsoon Forest* in the *Islands* exhibit. Orange lines signify approximate areas accessed by individual animals with dimensions of total available space. Copyright: Google Earth.

Throughout the pre-move data collection period, this species was housed in the *Monkey House* exhibit. Individuals had access to one indoor (120 m^2^ area) and one outdoor open-air ‘island’ area totalling 1496 m^2^ (Appendix B, Figure A3). Visitors had nearly 360° viewing opportunity for this species, particularly in the indoor area. The *Monkey House* exhibit included bark substrate flooring inside to prolong feeding behaviour and wooden poles connected with ropes and straps to encourage climbing. In the post-move data collection phase, this species was held in another part of *Monsoon Forest*, with access to one indoor enclosure (149 m^2^ area, 6258 m^3^ volume) and one open-air outdoor exhibit (2050 m^2^) (Appendix B, Figure A4). Species-specific features included multiple wooden poles connected with strapping, dynamic multi-level indoor space supplemented with climbable artificial rocks on the walls [32,33] and off-show areas where individuals could choose to be away from the view of visitors.

#### 2.2.3. Malayan Sun Bear Exhibit Information

**Figure A5:**
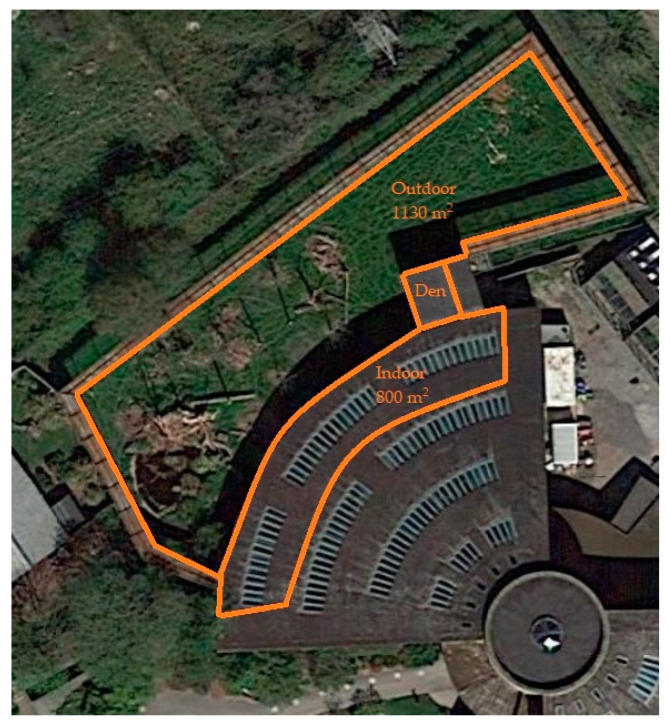
Enclosure plan for Malayan sun bear (*Helarctos malayanus*) in the *Spirit of the Jaguar* exhibit.Orange lines signify approximate areas accessed by individual animals with dimensions of total available space. Copyright: Google Earth.

**Figure A6:**
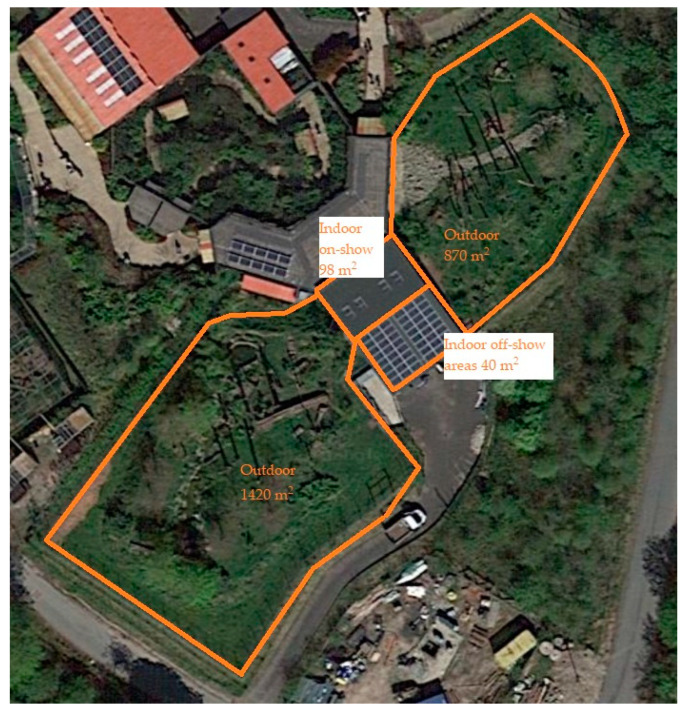
Enclosure plan for Malayan sun bear (*Helarctos malayanus*) in the new *Islands* expansion. Orange lines signify approximate areas accessed by individual animals with dimensions of total available space. Copyright: Google Earth.

Malayan sun bears were held in the *Spirit of the Jaguar* exhibit during the pre-move data collection phase. The savannah style of this exhibit comprised one indoor (800 m^2^) and one outdoor facility (1130 m^2^), with off-show den area access (two dens of 9 m^2^ each) (Appendix B, Figure A5). Wooden structures were included within the exhibit to facilitate climbing behaviour. The custom-built facility for Malayan sun bears in *Islands* included access to one indoor on-show area (98 m^2^), four indoor off-show den areas (8 m^2^ each totalling 32 m^2^), one off-show cubbing den (8 m^2^) and two on-show open air outdoor areas (1420 m^2^ and 870 m^2^) (Appendix B, Figure A6). Species-appropriate facilities include wooden poles of various heights to encourage climbing [34], a cubbing den, a dry ‘river-bed’ area with rocks providing a different substrate [35] and heavily planted zones to provide natural refuge sites from visitors.

#### 2.2.4. Malayan Tapir Exhibit Information

**Figure A7:**
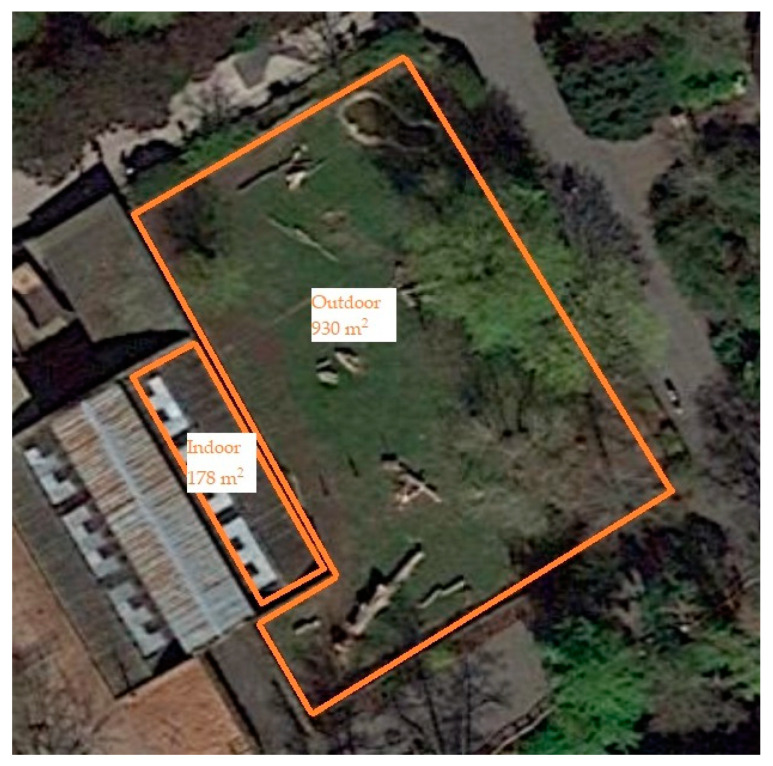
Enclosure plan for Malayan tapir (*Tapirus indicus*) in the ‘*Cattle House*’ exhibit. Orange lines signify approximate areas accessed by individual animals with dimensions of total available space. Copyright: Google Earth.

**Figure A8:**
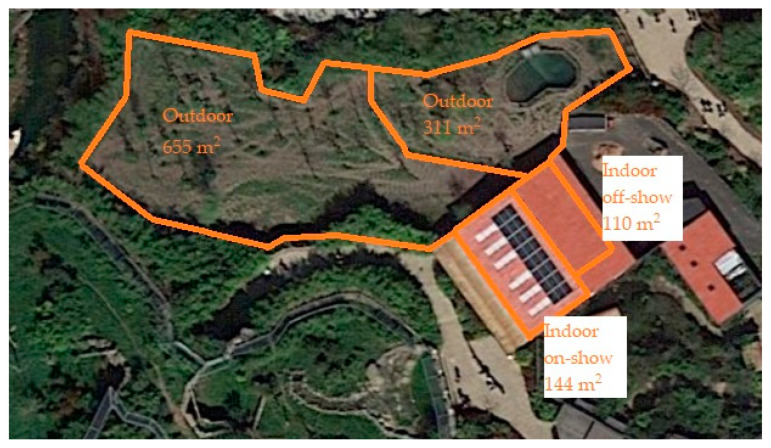
Enclosure plan for Malayan tapir (*Tapirs indicus*) in the new *Islands* expansion. Orange lines signify approximate areas accessed by individual animals with dimensions of total available space. Copyright: Google Earth.

Malayan tapirs were held in the *Cattle House* exhibit at Chester Zoo during the pre-move data collection condition. This area comprised indoor stall housing (178 m^2^ area) with an outdoor paddock (930 m^2^) (Appendix B, Figure A7). Individuals were provided with a shallow water area for bathing, but this pool was not deep enough for individuals to be fully submerged. Visitors had a 360° viewing opportunity for this species in the *Cattle House*. During the post-move condition, individuals had access to two indoor areas (one off-show: 110 m^2^, one on-show: 144 m^2^) and two outdoor open-air paddocks (655 m^2^ and 311 m^2^, including a 37 m^2^ pool area) (Appendix B, Figure A8). Species-appropriate facilities included two large pools for swimming, deep enough to allow full submersion, bark substrate flooring and live trees and shrubs in the outdoor paddocks to increase the complexity of the environment [36,37]. Individuals were able to move away from public viewing areas within this exhibit, with off-show pens and a dividing wall which allowed refuge from visitors.

### 2.3. Data Collection and Preparation

The data were collected by four observers for use in different sub-projects (one researcher per species). As such, data collection methods (including ethograms and sampling methods) varied by species: Sumatran orangutan and Malayan sun bear data were collected using continuous focal sampling, Malayan tapir data using group instantaneous scan sampling at 1 min intervals, and crested macaque data using focal individual instantaneous sampling at 30 s intervals [38]. All data were collected via live in-person observations during staff working hours between 08:30 and 17:00 using species-specific ethograms (Appendix A, Table A1, Table A2, Table A3 and Table A4). All observations were conducted at public viewing areas, with the researcher following subjects using these viewpoints throughout the observation session or until subjects entered an ‘off-show’ area which was out of sight. Risk assessment protocols were in place with contingencies outlined if the observer noticed that subjects were directly responding to observer presence, e.g., change clothing or abandon observation session. These contingencies were not utilized for any species within the study.

The length of observation sessions also differed between species: The majority of Sumatran orangutan observation sessions lasted approximately 30 min, yielding a median of 31 instantaneous samples per session (range: 4–320); Malayan sun bear observation sessions also lasted approximately 30 min, yielding a median of 30 instantaneous samples per session (range: 3–57); Malayan tapir observation sessions lasted approximately 60 min, yielding a median of 122 instantaneous samples per session (range: 61–244); crested macaque observation sessions lasted approximately 10 min, yielding a median of 11 instantaneous samples per session (range: 3–11).

To prepare these data for analysis and to enable comparison between species, all data were coerced into a consistent sampling format: focal individual instantaneous sampling at 1 min intervals. For example, the behavioural state was extracted every minute from continuous focal individual observations. These behaviours were then combined into 15 mutually exclusive categories that were/could be present in all 4 species (Table 2 and Appendix B).

**Figure 1:**
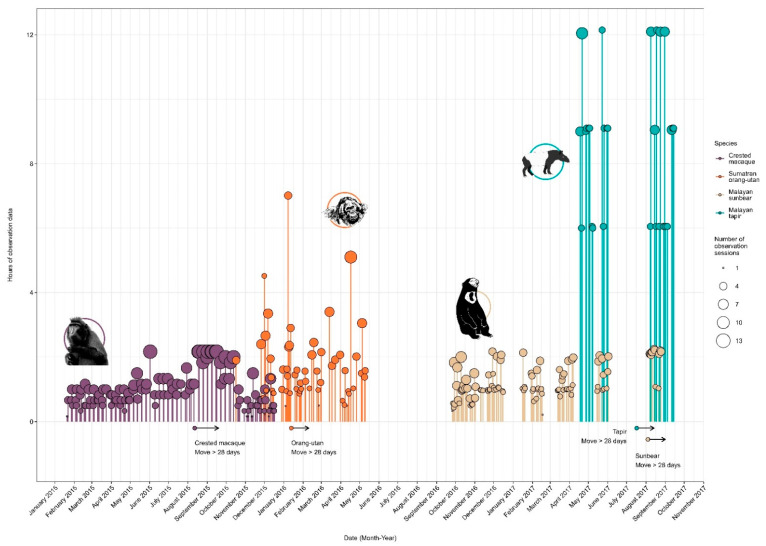
Data collection timeline and summary for four *Islands* project species: Sumatran orangutan (*Pongo abelii*), crested macaque (*Macaca nigra*), Malayan sun bear (*Heloarctos malayanus*) and Malayan tapir (*Tapirus indicus*) at Chester Zoo, UK. Stems and points indicate the number of hours of observation data and number of observation sessions per day, respectively. Arrows (reading from left to right) indicate the date of relocations to the new *Islands* exhibit from within the core zoo and subsequent 28 days (Early-Post). Data stems and points prior to the arrow signifies Pre-Move period and stems and points beyond the arrow signifies Post-Move periods.

These standardised data were organized into 3 conditions: ‘pre-move’ (collected over a 3–7.5 month range) when individuals were housed within the core zoo, immediately prior to their move; ‘early-post’ (the 28 days after transfer to new environments); and ‘post-move’ (collected over a 0.5–4.25 month range) after the initial 28-day period, when individuals were in their new environments. The ‘early-post’ condition was included so that any behavioural changes that occurred during an initial habituation/exploration period would be distinguishable from a longer-term response seen in the ‘post-move’ condition (see prediction P3 above). As the timeline for data collection varied between species, 28 days was chosen as the cut-off between the ‘early-post’ and ‘post-move’ condition to allow for a consistent evaluation of the immediate effect of translocation across all species. Additionally, data were plotted and inspected over time, revealing a more consistent behavioural response after the 28-day period, suggesting that individuals had settled into their new environment after this time. This yielded a total of 36,085 sampling points (representing 601 h and 25 min sampling time) over 290 observation days and 1255 observation sessions (Table 3 and Figure 1). Due to the purely observational nature of the research performed, no ethical approval for research was required. However, study methods and protocols were approved by Chester Zoo’s internal scientific review process.

### 2.4. Data Analysis

To investigate how the enclosure move influenced species’ behaviour, we examined the data across the three enclosure-move conditions in two different ways: (1) by comparing visible activity budgets (Activity budget approach: predictions P1 and P3) and (2) by comparing the proportion of the observation time spent out of sight of the observer/public (Choice and control approach: prediction P2). The four species were examined separately throughout the analysis, and all of our response variables were expressed as proportions of the total observation time. All data analysis was conducted in R (version 4.1.0) [39]).

#### 2.4.1. Activity Budget Approach (Models 1 to 4)

To investigate whether the enclosure move influenced species’ activity budgets, we fitted four linear mixed models (LMMs) with gaussian error distributions and identity links using the package ‘glmmTMB’ [40]. For our response variables, we calculated the proportions of time allocated to each behaviour described in Table 2, excluding not visible. For each individual, within each observation session (our sampling block/observational unit), we divided the number of instantaneous sample points recorded for each behaviour by the total number of sample points recorded. When an individual’s behaviour was recorded as other or not visible, these observations were discarded and subtracted from the total number of sample points.

Because activity budget data, by their nature, sum to 1 (or 100% if using percentages), the proportions of time spent in each activity are not independent. However, these types of data are habitually analysed as if each behavioural component were independent and unconstrained, e.g., by modelling the proportion of time spent performing each behaviour one-by-one, which can bias interpretation. When the response variable comprises more than two categories, this approach is inappropriate and can lead to spurious interpretation. Therefore, the activity budget data were analysed simultaneously using compositional models (Compositional Data Analysis (CoDA)). We transformed the proportional response variables using a centred log-ratio (CLR), which is the log of the ratio between the observed proportions of time spent performing each behaviour and their geometric mean per observation period. This removes the range restriction and allows for meaningful inference of the results of subsequent mixed models. Because the CLR transformation cannot handle zeros in the dataset, we first rescaled our proportions using the following formula, recommended for use when response variables are beta distributed [41]:

$$x^{\prime }=\frac{x\times \left(length\left(x\right)-1\right)+0.5}{length\left(x\right)},$$

where *x* is the observed proportion and *length*(*x*) our sample size.

We used the CLR-transformed proportions as compositional responses in our four by-species LMMs. To account for our first hypothesis (P1) that enclosure moves would influence activity budgets, we included as fixed effects behaviour (factor with up to 14 levels) and its two-way interaction with the enclosure-move condition (factor with 2–3 levels).

#### 2.4.2. Choice and Control Approach (Models 5 to 8)

To investigate whether the enclosure move influenced the proportion of time that species spent out of sight of the public/observer, we fitted two Beta general linear models (GLMs) and two Beta generalized linear mixed models (GLMMs) with logit links using the package ‘glmmTMB’ [40]. The Beta distribution is typically used to model continuous proportion data, and the logit link function ensures positive fitted values that range from 0 to 1 [42]. For our response variables, we calculated the proportions of time that each species spent out of sight. For each individual, within each observation session (our sampling block/observational unit), we divided the number of instantaneous sample points recorded as not visible by the total number of sample points recorded. Because these response variables comprised only two categories (out of sight vs. visible), Beta regression was used rather than CoDA: Beta regression models proportions at the original scale, which makes statistical inference simpler [43]. Prior to analysis, we rescaled our proportions using the formula detailed in Section 2.4.1, recommended for use when response variables are Beta distributed [41].

We used the not visible proportions as responses in four, by-species Beta regression models. We included as fixed effects the enclosure-move condition (factor with 2–3 levels), which accounted for our second hypothesis (P2) that enclosure moves would influence the proportion of time that species spent out of sight of the public/observer.

#### 2.4.3. Model Control Variables

All models included the same control variables: sex (factor with two levels: female, male) and age (continuous with range 281–10,749 days since birth). Age was scaled and centred prior to analysis. To incorporate the dependency among observations of the same individuals, and among observation sessions, the Sumatran orangutan and crested macaque models included crossed random intercepts for individual and observation sessions. Because the inclusion of random effects terms with fewer than 5 levels can destabilise mixed models, the Malayan tapir and Malayan sun bear models (three and four) only included the random intercept for the observation session, and for models seven and eight, no random effects were included. We weighted the four models with the total number of instantaneous sample points per species to account for the likely relationship between response accuracy and sample size.

#### 2.4.4. Model Assumptions and Additional Information

We used a full model approach throughout, and model fit and assumptions were verified by plotting residuals versus fitted values with the package ‘DHARMa’ [44]. We determined the significance of the fixed effects using likelihood ratio tests. We fitted full and restricted models (models in which the parameter of interest, the fixed effect, are withheld, i.e., fixed to 0) and based test statistics on comparisons of the full model with the restricted models. The significance of the likelihood ratio test statistic was calculated using a chi-squared distribution with the appropriate degrees of freedom. Where appropriate, post-hoc tests were carried out using Tukey’s HSD (honestly significant difference) tests, with the package ‘emmeans’ [45]. All statistical tests were two-tailed with α set to 0.05.

## 3. Results

### 3.1. Effect of the Enclosure Move on the Activity Budgets (Activity Budget Approach)

All full activity budget models differed significantly from their null model equivalents (Sumatran orangutan: χ2 = 30,434, degrees of freedom (df) = 37, *p* < 0.001; crested macaque: χ2 = 28,030, df = 28, *p* < 0.001; Malayan sun bear: χ2 = 43,683, df = 21, *p* < 0.001; Malayan tapir: χ2 = 145,634, df = 25, *p* < 0.001, Appendix C, Table A5, Table A6, Table A7 and Table A8). Similarly, the interaction between behaviour and enclosure-move condition was significant for all activity budget models (Sumatran orangutan: χ2 = 3665.3, df = 22, *p* < 0.001; crested macaque: χ2 = 1029.5, df = 16, *p* < 0.001; Malayan sun bear: χ2 = 1253.5, df = 9, *p* < 0.001; Malayan tapir: χ2 = 16,793, df = 14, *p* < 0.001), indicating that activity budgets differed between enclosure conditions (Appendix C, Table A5, Table A6, Table A7 and Table A8). To interpret the results of each model, we plotted the results and inspected the fitted values and their 95% confidence intervals throughout. Raw activity budget values can be found in the Appendix (Appendix C, Table A9).

#### 3.1.1. Model 1: Effect of the Enclosure Move on the Sumatran Orangutan Activity Budget

**Figure 2:**
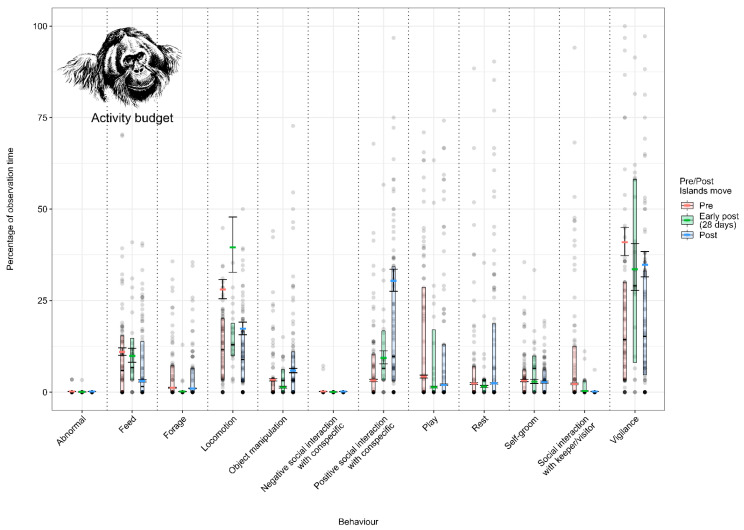
Proportion of time Sumatran orangutans spent performing each behaviour (expressed as a percentage of the total observation time) under the pre-, early-post- and post-enclosure-move conditions. The model’s fitted values are represented by coloured horizontal lines, and their respective 95% confidence intervals are depicted as black error bars. Coloured boxes with black horizontal lines depict the medians and quartiles of the response. Grey dots represent raw data points, shaded according to frequency (dark indicates many observations, light indicates few).

Sumatran orangutans spent less time feeding (from 11.0% (±1.8) to 7.5% (±1.2)) and locomoting (from 12.5% (±1.1) to 11 (±1.2)) post-move and more time engaged in object manipulation (from 4.9% (±1.0) to 8.7% (±1.5)) and positive social interactions with other orangutans (7.5% (±1.3) to 19.6% (±2.3)) (Figure 2; Appendix C, Table A9).

#### 3.1.2. Model 2: Effect of the Enclosure Move on the Crested Macaque Activity Budget

**Figure 3:**
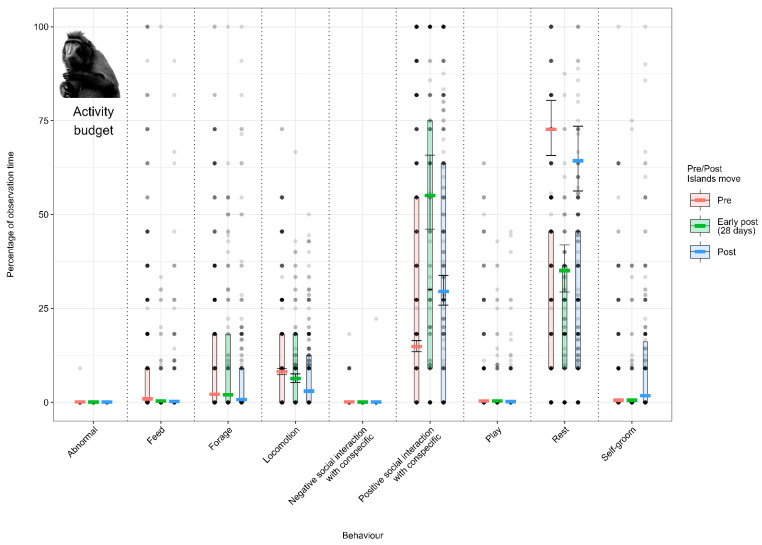
Proportion of time crested macaques spent performing each behaviour (expressed as a percentage of the total activity budget) under the pre-, early-post- and post-enclosure-move conditions. The model’s fitted values are represented by coloured horizontal lines, and their respective 95% confidence intervals are depicted as black error bars. Coloured boxes with black horizontal lines depict the medians and quartiles of the response. Grey dots represent raw data points, shaded according to frequency (dark indicates many observations, light indicates few).

Individuals spent less time feeding (from 9.2% (±1.0) to 4.3% (±1.0)) and locomoting (from 11.4% (±0.7) to 8.5% (±0.8)) between the pre- and post-move conditions. Additionally, macaques spent more time self-grooming (4.8% (±0.7), increasing to 10.5% (±1.3)) between the pre- and post-move condition (Figure 3; Appendix C, Table A9).

#### 3.1.3. Model 3: Effect of the Enclosure Move on the Malayan Sun Bear Activity Budget

**Figure 4:**
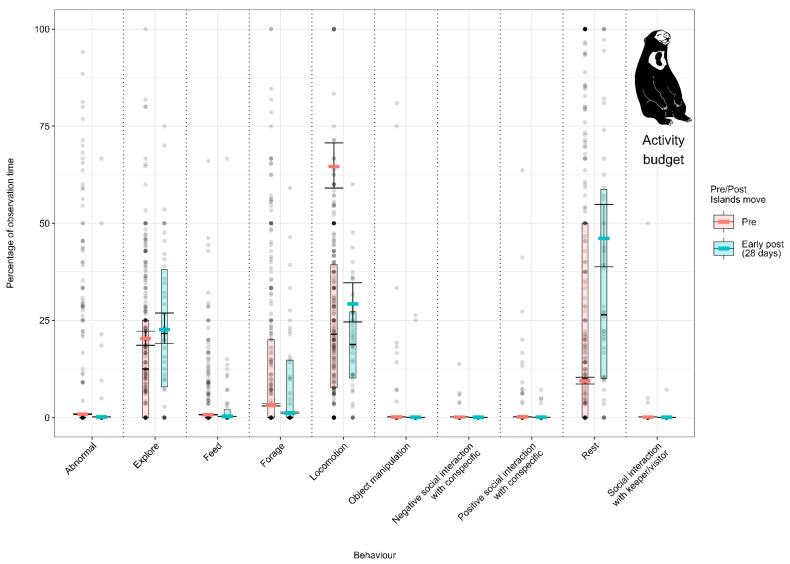
Proportion of time Malayan sun bears spent performing each behaviour (expressed as a percentage of the total observation time) under the pre-, early-post- and post-enclosure-move conditions. The model’s fitted values are represented by coloured horizontal lines, and their respective 95% confidence intervals are depicted as black error bars. Coloured boxes with black horizontal lines depict the medians and quartiles of the response. Grey dots represent raw data points, shaded according to frequency (dark indicates many observations, light indicates few).

Malayan sun bears spent less time foraging and locomoting and engaged in less abnormal behaviour in the 28 days post-move (Foraging: 13.3% (±1.4) to 9.2% (±2.2); Locomotion: 26.2% (±1.5) to 19.9% (±2.2); Abnormal behaviour: 9.6% (±1.3%) to 4.1% (±2.0)). Individuals spent more time resting under the early-post-move condition (37.2% (±5.0)) than pre-move (27.1% (±2.3)) (Figure 4; Appendix C Table A9). No data were available to estimate activity budgets after the initial 28-day early-post-move period.

#### 3.1.4. Model 4: Effect of the Enclosure Move on the Malayan Tapir Activity Budget

**Figure 5:**
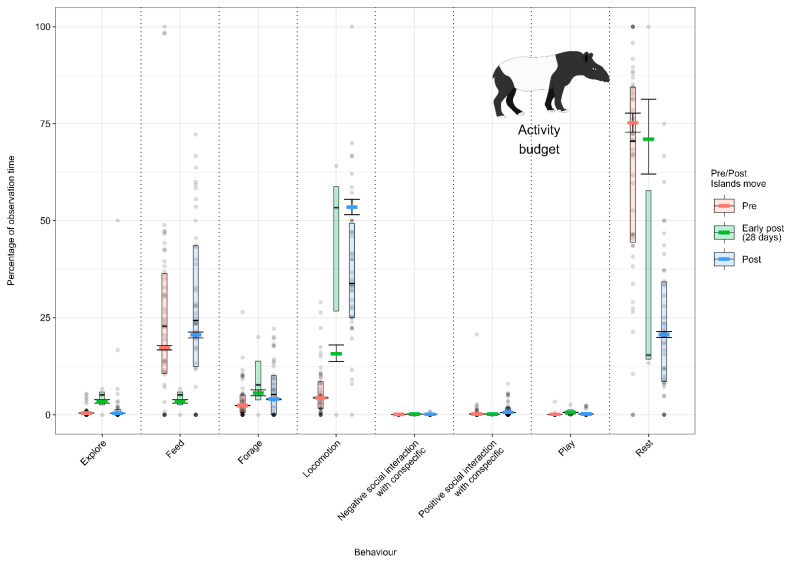
Proportion of time Malayan tapirs spent performing each behaviour (expressed as a percentage of the total observation time) under the pre-, early-post- and post-enclosure-move conditions. The model’s fitted values are represented by coloured horizontal lines, and their respective 95% confidence intervals are depicted as black error bars. Coloured boxes with black horizontal lines depict the medians and quartiles of the response. Grey dots represent raw data points, shaded according to frequency (dark indicates many observations, light indicates few).

From the pre- to post-move condition, individuals spent less time resting (62.7% (±3.9) to 23.3 (±2.9). Additionally, Malayan tapirs spent more time feeding (25.7% (±3.3) to 28.2% (±3.3)), foraging (3.8% (±0.7) to 6.7% (±1.0) and locomoting (6.2% (±0.9) to 37.9% (±3.0)) (Figure 5; Appendix C Table A9).

### 3.2. Effect of the Enclosure Move on the Visibility (Choice and Control Approach)

Overall, the null model significantly differed from the full model for crested macaques (χ2 = 30.202, df = 4, *p* < 0.001, Model 6), Malayan sun bears (χ2 = 20.923, df = 3, *p* < 0.001, Model 7) and Malayan tapirs (χ2 = 89.09, df = 4, *p* < 0.001, Model 8), indicating that the move to a new environment influenced the visibility of these species in public viewing areas. However, the null model did not significantly differ from the full model for Sumatran orangutans (χ2 = 1.581, df = 4, *p* = 0.812, Model 5), suggesting that visibility was not influenced by a move to a new environment for this species.

#### 3.2.1. Model 5: Effect of the Enclosure Move on the Sumatran Orangutan Visibility

**Figure 6:**
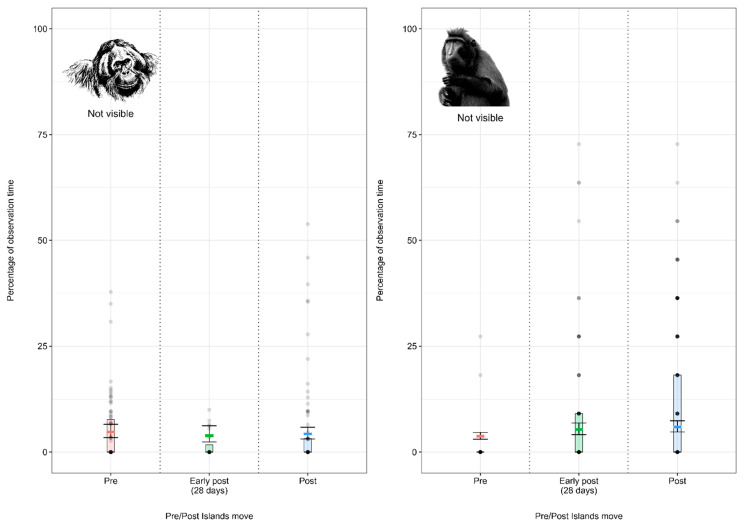
Proportion of the observation time (expressed as a percentage of the total observation time) Sumatran orangutans and crested macaques spent out of sight of observers/public under the pre-, early-post- and post-enclosure-move conditions. The model’s fitted values are represented by coloured horizontal lines, and their respective 95% confidence intervals are depicted as black error bars. Coloured boxes with black horizontal lines depict the medians and quartiles of the response. Grey dots represent raw data points, shaded according to frequency (dark indicates many observations, light indicates few).

Sumatran orangutans spent similar proportions of time out of sight of observers/public before, immediately after (28 days) and following the enclosure move (Figure 6; Appendix C Table A10).

#### 3.2.2. Model 6: Effect of the Enclosure Move on the Crested Macaque Visibility

Crested macaques spent significantly more time out of sight of observers/public immediately after (28 days) and following the enclosure move (Figure 6; Appendix C Table A11) than pre-move.

#### 3.2.3. Model 7: Effect of the Enclosure Move on the Malayan Sun Bear Visibility

**Figure 7:**
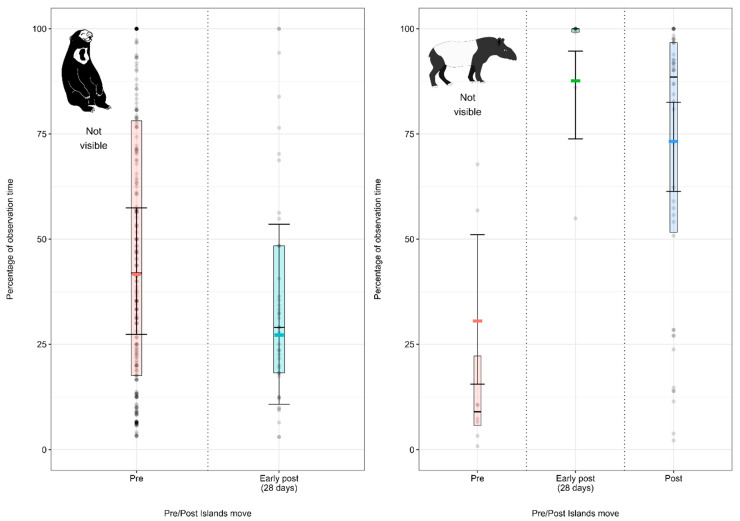
Proportion of observation time (expressed as a percentage of the total observation time) Malayan sun bears and Malayan tapirs spent out of sight of observers/public under the pre-, early-post- and post-enclosure-move conditions. The model’s fitted values are represented by coloured horizontal lines, and their respective 95% confidence intervals are depicted as black error bars. Coloured boxes with black horizontal lines depict the medians and quartiles of the response. Grey dots represent raw data points, shaded according to frequency (dark indicates many observations, light indicates few).

Malayan sun bears spent less time out of sight of observers/public in the 28 days post-move than before the enclosure move (Figure 7; Appendix C Table A12).

#### 3.2.4. Model 8: Effect of the Enclosure Move on the Malayan Tapir Visibility

Malayan tapirs spent more time out of sight of observers/public following the move (in the 28 days post-move and thereafter) than before the enclosure move (Figure 7; Appendix C Table A13).

## 4. Discussion

Activity budget comparison during a management or environmental change is often used to assess whether an intervention has influenced animal behaviour [3,23,26,46]. Here, we presented activity budgets for four species during three periods of data collection: pre-move, early-post and where available, post-move. In line with our first prediction (P1), the move to a new environment significantly affected the activity budget of all species. Furthermore, the visibility of three of the four species to members of the public was also influenced from pre-move to post-move as predicted (P2). Additionally, in support of prediction 3 (P3), we found evidence that activity budgets differed significantly between the early-post and post-move conditions, indicating that there was a period of habituation to the new environment. We show here the value of presenting and considering full activity budgets when evaluating the influence of an environmental change, as it allows an examination of the relative change in the proportions of expressed behaviour.

### 4.1. Activity Budget Approach

Our study revealed consistent behavioural responses across study species. For example, abnormal behaviour remained low, absent or declined in all four species. The absence of abnormal, repetitive behaviours is generally used as an indicator of positive welfare [47]. However, because some of these behaviours can be “learned” in potentially sub-optimal previous enclosures (and so may not reflect current welfare experience), the use of abnormal behaviour as a measure of enclosure suitability should be interpreted with caution [48,49]. Additionally, an absence of abnormal behaviour does not necessarily mean that the animal’s welfare is optimal [2]. Despite this, zoos often strive to reduce or eliminate abnormal behaviours by encouraging the expression of species-typical behaviour through good husbandry practices [50,51], naturalistic feeding regimes [26,52] and/or enhancing enclosure environments [52].

Our results also indicated a consistent increase in the expression of positive social behaviour with conspecifics for both primate species from pre- to post-move. Positive social interactions such as allogrooming and play are vital for social cohesion and the maintenance of group structure in many primates [53,54], with investment in this behaviour often prioritized after periods of disturbance [55]. Allogrooming can function as a form of stress prevention (social buffering) in crested macaques, with reductions in self-directed behaviour and aggressive tendencies observed after grooming sessions [56]. Similarly, observing allogrooming may also promote the expression of other positive social behaviours (e.g., *Macaca sylvanus* [53]), highlighting the importance of these behaviours at the individual and group levels. Despite this, research into the effects of translocation on pro-social behaviour is limited. However our findings are in line with those reported by Schaffner and Smith [56], that Wied’s marmosets (*Callithrix kuhlii*) tend to seek a partner’s proximity more after an enclosure move than before. From pre- to post-translocation, grooming rates fell among tufted capuchin monkeys (*Cebus apella*), but the mean number of grooming partners increased [57]. Our work adds to the body of literature highlighting the importance of maintaining species-appropriate social groups during translocation events, as they have the potential to play a key role in both individual and group level welfare.

#### 4.1.1. Sumatran Orangutan Activity Budget

Individuals were observed engaging in less feeding and more locomotor behaviour following the exhibit move. The decrease in time spent feeding from the pre- to post- move condition could be attributed to individuals investing more time in maintaining group social relationships over time [58], whilst the increase in locomotion early-post-move is likely an exploratory response to the novel environment. Social interaction with humans decreased from pre-move levels of 10.2% (±2.0) of the activity budget to 0.1% (±0.1) post- and early-post move. Visitors can be a source of stressful excitement for zoo-housed primates [59], with reported increases in activity correlating with visitor number [60]. When we examined which category of humans the orangutans interacted with (keepers vs. visitors), we found that the reduction in time spent interacting with humans was largely driven by a reduction in time spent interacting with visitors (6.5% pre-move to 0.0% post-move). Notably, two hand-reared female orangutans were responsible for the majority of the visitor interactions, highlighting the importance of early-life-history context in assessing welfare. Visitors tend to perceive interactions with orangutans as positive experiences (K.Finch, pers comms); however, it is important to provide animals with the opportunity to avoid such interactions, and thoughtful enclosure design can enable this [61]. Additionally, individuals still had opportunities and areas to engage with visitors in their new environment, indicating that during both the early-post and post-move conditions, individuals were choosing to engage in other behaviours elsewhere. Orangutans often use and manipulate objects as tools [62]. This species-specific behaviour, defined here as ‘object use’, increased following the move to the new enclosure; from 3.4% of the activity budget pre-move to 7.3% post move. Novel planting and vegetation were available in the new environment, and these were often used to reach other resources within the area. Encouraging the expression of species-appropriate behaviour in zoo-housed individuals is essential in the optimization of animal well-being [63], and thus categorization of these behaviours is important in the evaluation of the welfare state.

#### 4.1.2. Crested Macaque Activity Budget

Crested macaques spent less time feeding and locomoting, but more time engaged in positive social behaviour and autogrooming following the enclosure move. Self-directed behaviours in primates such as scratching, self-grooming and face touching are associated with physiological and psychological arousal (often stress) [64,65,66]; however, such gestures may also serve a communicative function [67]. Group members may gain insight into an individual’s motivational state by observing their self-directed behaviours, such as self-grooming, allowing them to better mediate their social interactions [68]. However, the increase in self-grooming was accompanied by an increase in positive social interaction with conspecifics, suggesting that affiliative social bonds between individuals may mediate some of the stressful effects associated with a period of uncertainty [69]. Upon comparison to pre-move values, it was observed that positive social behaviour increased during the early-post-move condition whilst autogrooming increased during the post-move condition. The difference in the latency of responses could be due to the adoption of both short- and long-term coping strategies to the move to a new environment by this highly social species. Macaques also spent less time resting immediately following the enclosure move (during the early-post-move condition), but the percentage of time spent resting returned to pre-move levels after the initial four weeks had passed. A shift towards more active behaviours during the early-post-move phase could be attributed to a process of habituation to the novel environment, with similar patterns observed in both zoo-housed [70] and wild primates [71]. Our results show that species natural history should be taken into consideration when evaluating the influence of an exhibit move on behaviour. For highly social species such as the crested macaque, the social dynamic and group composition may be more influential to overall activity budget than environment complexity or naturalism.

#### 4.1.3. Malayan Sun Bear Activity Budget

Law & Reid [62] highlight the importance of appropriate enclosure design for zoo-housed bear species, outlining that good husbandry practice, including a variety of targeted enrichment techniques, should work in harmony with well-designed architecture to optimize bear welfare.

Malayan sun bears were generally less active following the enclosure move in this study. Individuals spent less time foraging and locomoting and more time engaged in resting behaviour between the pre-move and post-move condition. Additionally, individuals reduced their time exhibiting abnormal behaviours by 5.5% from pre- to post-move. Malayan sun bears housed in enriched outdoor environments showed a similar pattern of behaviour [72], with increased resting and reduced stereotypy compared to bears kept in more barren environments. Bears are known to exhibit a range of abnormal repetitive behaviours in zoos [62], with management practices, such as feed regimes, and previous experience, i.e., mother or hand-reared, found to be directly linked to stereotypy occurrence [73]. Both individuals in this study were bought into captivity as a consequence of the illegal wildlife trade (T Rowlands, pers comms); therefore, this could have some influence on the behavioural repertoire observed during the study. Exhibit size and complexity have also been associated with behavioural and physiological benefits in polar bears (*Ursus maritimus*), showing that larger land areas and the ability for individuals to view out of their exhibit resulted in lower stereotypy engagement [74]. The outdoor environment in the new *Islands* exhibit is larger for this species than the previous facility, and this combined with species-appropriate features such as wooden climbing poles and varying environmental substrates (Appendix B, Figure A6) could have influenced the behavioural changes observed from pre- to post-move. However, it must be taken into consideration that our sun bear data only cover the pre- and early-post-move conditions; as such, these behavioural responses could be a short-term response to a new environment. For example, explorative behaviour increased by 6.6% during the early-post-move condition, suggesting that individuals spent time familiarizing themselves with the novel environment.

#### 4.1.4. Malayan Tapir Activity Budget

Active behaviour increased following the enclosure move for Malayan tapirs. Recent work on zoo-housed Malayan tapirs is limited; however, activity budgets for zoo-housed South American tapirs (*Tapirus terrestris*) mainly comprise rest, feeding, foraging and locomotion [75]. We found that tapirs spent more time feeding and foraging following the enclosure move. This is largely consistent with the findings of Arumugam et al. [76], who highlighted that enclosure type had a significant influence on feeding behaviour for this species, with increased feeding observed in naturalistic vs. artificial environments. This suggests a measure of success for the *Islands* expansion as the tapir enclosure was designed to be more naturalistic and environmentally complex than their previous enclosure, with the specific aim of facilitating the expression of natural active behaviours. Prior to the enclosure move, the tapirs spent the majority of their time resting (62.7% (±3.9) of their activity budget). Following the enclosure move, this reduced by 39.4% to 23.3% (±2.9). However, this decrease should be interpreted with caution as the tapirs were also considerably less visible post-move, and it is plausible that resting time remained consistent but unrecorded. Changes in activity levels have also been reported in several other species after a move to a new environment [10,77,78,79,80,81], and it is possible that any decrease in time spent resting could be attributed to exposure to a novel area, i.e., time spent exploring increases at the expense of rest. However, by separating our analyses into three blocks (one of which should encompass this habituation/exploration period; early-post-move), we can be largely confident that the post-move behaviour changes represent a longer-term response. For example, although an increase in non-repetitive locomotor behaviour may be attributable to an individual exploring their new habitat [77], care should be taken to record if this behaviour becomes excessive or follows a specific route (route-tracing), as this could be indicative of locomotor stereotypy [82,83]. When we examined which behaviours comprised ‘Locomotion’, we found that swimming increased from 0.05% of the activity budget to 4.7% following the move to the *Islands* enclosure. Tapirs are one of few semi-aquatic hoofed animals, thus access to an appropriate water source is suggested to be essential for their welfare [84]. The pool in the pre-move enclosure was not deep enough for individuals to fully submerge and swim, and this was key in the design of the new enclosure. As such, the *Islands* enclosure contained two larger pools (Appendix B, Figure A8). The expression of swimming behaviour for this species provides a clear example of the benefits and importance of considering species’ natural behaviour during the enclosure design process.

### 4.2. Choice and Control Approach

Modern animal welfare assessment highlights the importance of allowing individuals to exercise a level of choice and control over their environment [20]; for zoo animals, this includes the amount of time that individuals spend in public view. Our study revealed that for three of the four species studied, the move to a new environment influenced the time that individuals spent visible to the public. Malayan sun bears spent more time in public viewing areas immediately following the move (early-post-move condition). Conversely, crested macaques and Malayan tapirs spent less time visible to the public following the enclosure move. Both species had little choice in their pre-move environments regarding being visible to the public. However, once given the facilities in their new environment to be out of public view, our data showed that the crested macaques and Malayan tapirs utilised these areas. The transition to a new environment did not influence the amount of time spent in public viewing areas for Sumatran orangutans.

Research into human-animal relationships (HARs) has increased in the last decade with a variety of positive, neutral and negative welfare outcomes reported from HARs in zoo-settings [85,86,87,88,89,90,91,92]. Visitors play an important role in this dynamic, with many expressing their enjoyment at observing natural behaviour and learning about zoo-housed species, yet visitors often state they want to do so in close proximity to the animals themselves [52]. This presents an interesting conflict of interest for zoo managers, as there is increasing evidence outlining the behavioural and welfare benefits of giving animals a level of agency within their environment and daily lives [93]. Ritzler et al. [94] outlined that when provided with environmental choice, individuals increased locomotor and other active behaviours compared to when they were restricted. Furthermore, Ross [95] reported that access to off-exhibit holding space decreased stereotypy and increased social play in a pair of captive polar bears (*Ursus maritimus*). When investigating the relationship between animal behaviour and visitor perception at a Chinese zoo, animal presence elicited a similar visitor response at both naturalistic and barren exhibits. However, visitor interest persisted at the naturalistic exhibit even when animals were not visible [96]. Given that enclosures with a naturalistic design are suggested to provide a more suitable environment for zoo-housed species [97], institutions may be able to ensure optimal animal welfare by allowing them to exert a level of choice and control over their environment, whilst maintaining visitor satisfaction.

A potential limitation of this research was controlling for other factors which may affect behaviour such as weather [98], seasonality [99,100,101], observer influence [102,103] and visitor presence [96,104,105]. Any future research should consider collecting these variables. Remote behavioural monitoring through networked cameras has been shown to provide a non-invasive, observer-free insight into behaviour through periods of change for zoo-housed species [106] and so should be considered if possible. However, the interaction between these variables must also be examined when considering the influence they may have on behaviour. Goodenough et al. [107] outlined that both time and weather can lead to the overestimation of visitor effects, a finding which needs to be considered for when future work is conducted in this area.

## 5. Conclusions

In line with our first prediction (P1), the move to a new environment significantly altered engagement in pre-determined behavioural categories for all four species studied. Using compositional data analysis, we were able to investigate the relative change in the proportion of certain behaviours over time following the move to a more naturalistic environment. In line with our second prediction (P2), we revealed that time spent in areas visible to the public changed for three of the four species following translocation. Finally, we highlight the importance of including a habituation period when conducting behavioural observations during the evaluation of these management events. In line with our final prediction (P3), engagement in certain behavioural categories did change across all species between the early-post and post-move condition, indicating individuals had a period of time in which they transitioned and adapted to their new areas. By gaining insight into the proportion of time spent in specific behaviour categories using activity budgets and compositional data analysis, we can infer that the new environments provided in the *Islands* expansion are successful in facilitating species-appropriate behaviours whilst still providing viewing opportunities for visitors.

## Figures and Tables

**Table 1:** Study species, subjects and data collection periods. Females represented by ♀, males represented by ♂.

Species	Adult		Sub-Adult		Moving Date (Y-M-D)	Pre-Move Data Collection	Post-Move Data Collection
	♀	♂	♀	♂			
Sumatran orangutan	3	1	1	1	13 January 2016	17 October 2015 to 12 January 2016	19 January 2016 to 10 May 2016
Crested macaque	8	3	2	0	12 August 2015	14 January 2015 to 11 August 2015	18 August 2015 to 17 December 2015
Malayan sun bear	1	1	0	0	4 August 2017	21 November 2016 to 2 June 2017	7 August 2017 to 31 August 2017
Malayan tapir	1	1	1	0	17 July 2017	18 April 2017 to 1 June 2017	8 August 2017 to 14 September 2017

**Table 2:** Descriptions of behaviours used for analysis.

Behavioural Category	Cross-Species Definition
Abnormal	Any repetitive, unnatural, or stereotypical behaviours that show no obvious goal or function to the individual: Includes pacing, window licking, over-grooming, self-directed aggression, head swaying/throwing.
Autogroom	An individual picking, licking, scratching, biting, rubbing, or slow-brushing their own body.
Explore	Investigating and/or searching for things inside the enclosure: includes sniffing, scratching, staring and digging.
Feed	Ingesting food.
Forage	Actively searching for and/or manipulating food.
Interaction with humans	Move and/or direct attention towards human(s): Includes keepers and visitors and often involves eye-contact.
Locomotion	Moving from one place to another: Includes climbing, swimming and bi/quadrupedal movement.
Object manipulation	Actively or passively touching, playing with, sniffing, or otherwise engaging with an object: Includes nest-building and engagement with enrichment items/puzzle feeders.
Other	Any behaviour not subsumed in the other categories: includes excretion and vocalisation.
Play	Excitable, non-aggressive, playful behaviour: includes running, pursuit, mock-fighting, bucking and leaping. Can be alone or with another individual.
Rest	Individual is observed sitting, lying, or standing without movement.
Negative social interaction with conspecific	Aggressive and/or threatening behaviour between individuals. Includes slapping, hitting, displacement, charging and antagonistic facial expressions.
Positive social interaction with conspecific	Affiliative behaviour between individuals: Includes allogrooming, maternal behaviour, contact-sitting, nuzzling, mating and affiliative facial expressions.
Vigilance	Individual is observing/scanning and aware of surroundings; eyes and/or head in movement.
Not visible	Individual is not visible from observer’s viewing position.

**Table 3:** Number of behavioural sampling points used for analysis by species and enclosure-move condition.

Species	Number of Sampling Points (Hours:Minutes)			
	Pre-Move	Early-Post	Post-Move	Total
Sumatran orangutan	2894 (48:14)	678 (11:18)	2594 (43:14)	6166 (102:46)
Crested macaque	3916 (65:16)	1111 (18:31)	2046 (34:06)	7073 (117:53)
Malayan sun bear	6762 (112:42)	1459 (24:19)	0 (0:0)	8221 (137:01)
Malayan tapir	7305 (121:45)	1098 (18:18)	6222 (103:42)	14,6225 (243:45)
Total	20,877 (347:57)	4346 (72:26)	10,862 (181:02)	36,085 (601:25)

## Data Availability

Data available upon request.

## Appendix A

**Table A1:** Sumatran orangutan (*Pongo abelii*) sub-project ethogram.

Behaviour	Definition	Combined Behaviour Category
Alert	Observing and aware of surroundings; if face cannot be seen, head is visibly moving.	Vigilance
Affiliative social interaction	Other social behaviour not outlined which maintains or improves group cohesion, can include food/resource sharing or non-maternal carrying from another individual.	Positive social interaction with conspecifics
Agonistic social interaction	Behaviour which reduces group cohesion, can include slapping, biting, wrestling or grabbing. Also includes non-contact interaction such as displacement, lunging or chasing.	Negative social interaction with conspecifics
Being groomed	Having fur picked through or gently scratched by another orangutan.	Positive social interaction with conspecifics
Consumption	Actively drinking, chewing or eating food.	Feed
Excretion	The elimination of urine or faeces from the body.	Other
Foraging	Searching for food, including taking berries from plants, and using fingers and/or mouth to retrieve food from mesh after a scatter feed.	Forage
Grooming others	Picking through or gently scratching the fur of another orangutan.	Positive social interaction with conspecifics
Grooming self	Picking through fur or teeth, scratching and rubbing against objects to try to clean skin or hair.	Self-groom
Keeper interaction	Looking towards or placing objects or limbs into the keeper areas.	Social interaction with keeper/visitors
Locomotion	Moving from one point to another within the exhibit, can include the use of enclosure furnishings such as ropes, nets, sway poles. Includes bipedal and quadrupedal locomotion.	Locomotion
Maternal behavior	Actions related to the care of an infant, includes cradling, nursing or carrying.	Positive social interaction with conspecifics
Nest building	Manipulation of materials within exhibit to form a nest.	Object manipulation
Not visible	Individual and its behaviour cannot be seen by observers.	Not visible
Play	Excitable behaviour which has no specific outcome or objective, can include playful chasing, wrestling or swinging on ropes with another individual.	Play
Resting	Sp. is relaxed and displaying no alert or active behaviours, eyes can be open or closed.	Rest
Sexual	Includes heterosexual mounting and copulation, the presentation of the anogenital region to another individual or the inspection of the anogenital area to solicit mating.	Positive social interaction with conspecifics
Solitary play	Excitable behaviour which has no specific outcome or objective, includes swinging on ropes and interacting with enclosure resources in a playful manner.	Play
Tool use	Utilisation of objects within the environment to achieve a specific task.	Object manipulation
Visitor interaction	Sitting by or looking through visitor windows, includes reacting to visitors by banging the glass using fists or objects within the enclosure.	Social interaction with keeper/visitors
Vocalising	Exhibiting one or a combination of calls which could include a kiss squeak, long call, whimper or rolling call.	Other

**Table A2:** Crested macaque (*Macaca nigra*) sub-project ethogram.

Behaviour	Definition	Combined Behaviour Category
Active	Behaviours in which individuals are travelling slowly (walking) or quickly (running). This behavior can include vertical travelling (climbing).	Locomotion
Agonistic	An individual chases, slaps, grabs, hits, or bites another individual. This behavior is frequently accompanied with open mouth, bared teeth screams in which the mouth is open wide with corners retracted, exposing the teeth and sometimes the gums. This facial expression is accompanied by staring and screaming vocalizations.	Social negative
Allogrooming	An individual picking or slowly brushing the fur of another individual using the hands or mouth.	Social positive
Autogrooming	An individual picking or slowly brushing their own fur using the hands or mouth.	Autogroom
Away from public view	Individual is not visible from the public viewing areas.	Not visible
Contact-sit/Mutual embrace	An individual passes one or both hands, or one or both arms around the body of another in multiple combinations. There may be a simultaneous grasping of the fur and contact between heads or chests. Embracing may be mutual or unilateral. This behavior may be accompanied by lipsmacking.	Social positive
Environmental play	Individuals are observed engaged in play behavior with their physical surroundings, for example, with branches, mesh, or ropes. This behavior is distinguished from environmental manipulation by the absence of violent shaking of an object.	Play
Feeding	An individual ingesting food or water. Food ingestion is accompanied by chewing.	Feed
Foraging	Individuals moving substrates with their hands in search for food.	Forage
Hold-bottom	An individual clasps the haunches of a partner with both hands and grasps its legs with feet as in a mount.	Social positive
Huddle	Groups of ≥ three individuals embracing.	Social positive
Lipsmacking	The lips are pursed, and the lower jaw is moved up and down rapidly and rhythmically. The jaw may be thrust upward. The mouth may be slightly open with the tongue moving back and forth. The lips often produce an audible sound. Alternatively, the mouth may be closed, and sometimes the teeth knock together. Eyelids are generally half-lowered. The scalp may be retracted and the ears flattened. The display is used during affiliative interaction. It may also end a conflict and acts as an appeasement or reassurance signal.	Social positive
Mating	An individual climbs ventrodorsally upon a standing partner and inserts his erect penis in the female’s genitals. The mounter may or may not grip the legs of the partner.	Social positive
Play	An individual contacts another in the context of play. This may include touching, slapping, bumping, jostling, pushing, grasping, catching, chasing, pulling, nibbling, dragging, lifting, climbing or leaping over the partner, along with other patterns possibly occurring outside the context of play (e.g., mount, mouth approach). These patterns are accompanied by silent bared-teeth facial expressions. Play behavior is distinguished from conflict by a lack of loud vocalizations.	Play
Resting	Individual is observed sitting, lying, or standing without movement. Individuals are not interacting with others, but may be in physical contact with them.	Rest
Silent bared-teeth	The upper lip or both lips are vertically retracted, exposing the teeth and sometimes the gums. The corners of the mouth may be drawn back. The jaw may be either closed or opened to various degrees. The scalp is often raised and the ears flattened. This is an affiliative display; it is commonly observed during affiliative interactions and social play.	Social positive
Window licking	An individual is observed licking the glass-viewing panels.	Abnormal

**Table A3:** Malayan sun bear (*Helarctos malayanus*) sub-project ethogram.

Behaviour	Definition	Combined Behaviour Category
Charging	Swift and firm movement directly at a conspecific.	Social negative
Climbing	Vertical locomotion up or down tree/post or up and over logs.	Locomotion
Consumption	Voluntary ingestion of edible material and liquids.	Feed
Elimination	Elimination of urine and faeces from body.	Other
Foraging	Digging and manipulating objects in search of edible material.	Forage
Grooming	Includes licking, scratching, biting and rubbing of own body.	Autogroom
Interaction	Non-aggressive interaction with a conspecific; includes sniffing, leaning, nuzzling and grooming.	Social positive
Interaction with keeper(s)	Move towards and/or stands on hind limbs while facing the keeper(s), often with eye contact.	Interaction with humans
Interaction with visitor(s)	Move towards and/or stands on hind limbs while facing the visitor(s), often with eye contact.	Interaction with humans
Manipulation	Manipulation of object(s), e.g., branches, to create a nest.	Exploratory
Mating	Mounting or attempting to mount a conspecific.	Social positive
Not visible	Entire body is out of view.	Not visible
Other	Behaviour not described in ethogram.	Other
Other stereotypy	Repetitive behaviour including head sway/throw, foaming, pica, regurgitating, self-directed aggression and over-grooming, including licking, sucking, fur-plucking.	Abnormal
Pacing	Continuous walking back and forth in a repetitive way at least three times. Also includes forward–reverse pace (without turning the body) and weaving.	Abnormal
Playfighting	Includes non-aggressive pursuit, gentle/playful wrestling, biting and jawing (mouth to mouth in a playful context).	Play
Rest—awake	Sitting, lying, or leaning so that part or all of body is supported, while appearing attentive with head raised in air.	Rest
Rest—sleep	Sitting, lying, or leaning so that part or all of body is supported, with body motionless and head resting; does not appear alert.	Rest
Retreating	Walking or running away from a conspecific that is showing aggressive behaviour.	Social negative
Running	Horizontal locomotion where there is a period of time during each stride in which no limbs are in contact with the ground.	Locomotion
Sniffing	Briefly inhaling an object, ground, or air during olfactory investigation.	Exploratory
Standing	Maintaining an upright position on extended legs, with equal distribution of weight bipedally or quadrupedally.	Rest
Threatening	Snout wrinkled upwards with mouth open, showing canines, and often vocalising loudly.	Social negative
Unknown	Part or the body is obscured making accurate identification of behaviour impossible.	Other
Vocalisation	Opening the mouth and producing sound. May occur while solitary, at a conspecific, or at human(s). Not to be confused with threatening.	Other
Walking	Horizontal locomotion where at least one limb will be in contact with the ground at any given time.	Locomotion

**Table A4:** Malayan tapir (*Tapirus indicus*) sub-project ethogram.

Behaviour	Definition	Combined Behaviour Category
Aggression Given	Displaying aggressive behaviours such as biting and chasing shown to any other individual.	Social negative
Aggression Received	Any other individual displaying aggressive behaviours such as biting and chasing towards the focal animal.	Social negative
Defecation	Excreting faeces.	Other
Drinking	Ingesting water.	Feed
Eating	Ingesting food.	Feed
Foraging	Walking whilst actively searching for food on the ground and ingesting it.	Forage
Locomotion (run)	Moving around the enclosure at a fast pace.	Locomotion
Locomotion (walk)	Moving around the enclosure at a “normal” walking pace.	Locomotion
Not Visible	Out of sight of the researcher.	Not visible
Other	Any behaviour that is not listed on the ethogram.	Other
Play	Excitable behaviour often including running, bucking and vocalisation, can be shown alone or play with another individual.	Play
Resting (Lying Down)	Stationary with body on the ground, eyes open or closed with head moving or still.	Rest
Resting (Sitting)	Upright with hind legs on the ground, eyes open or closed with head moving or still.	Rest
Standing	Stationary in an upright standing position–potentially alert or resting.	Rest
Stereotypies	Any repetitive, unnatural behaviours that show no obvious goal or function to the individual.	Abnormal
Sniffing	Any visible movement of the nose excluding the flehmen response.	Exploratory
Social Interaction	Any behaviour shown between individuals including social grooming and contact.	Social positive
Suckling	When the juvenile tapir is feeding from the mother.	Social positive
Swimming	In water or pool (note: this behaviour may only be possible in the new enclosure).	Locomotion
Urination	Excreting urine.	Other
Vocalisation	Making audible sounds.	Other

## Appendix C

**Table A5:** Model 1 summary output: Sumatran orangutan activity budget.

Term	Estimate	SE	Lower CI	Upper CI
(Intercept)	−2.778	0.048	−2.871	−2.684
Behaviour:				
(autogroom)	2.862	0.066	2.733	2.99
(feed)	4.108	0.066	3.979	4.236
(forage)	1.894	0.066	1.765	2.023
(locomotion)	5.045	0.066	4.916	5.174
(object manipulation)	2.924	0.066	2.795	3.052
(play)	3.155	0.066	3.026	3.283
(rest)	2.568	0.066	2.439	2.696
(social human)	2.515	0.066	2.387	2.644
(social negative)	−0.04	0.066	−0.169	0.088
(social positive)	2.878	0.066	2.749	3.007
(vigilance)	5.425	0.066	5.296	5.553
Enclosure-move condition:				
(early-post-move)	−0.136	0.107	−0.345	0.073
(post-move)	0.04	0.068	−0.092	0.173
Sex:				
(female)	0	0.02	−0.038	0.038
Age (days)	0	0.009	−0.018	0.018
Behaviour * Enclosure-move condition:				
(autogroom * early-post-move)	0.632	0.151	0.336	0.927
(feed * early-post-move)	0.622	0.151	0.327	0.917
(forage * early-post-move)	−1.358	0.151	−1.653	−1.062
(locomotion * early-post-move)	1.075	0.151	0.780	1.371
(object manipulation) * early-post-move)	−0.176	0.151	−0.471	0.120
(play * early-post-move)	−0.392	0.151	−0.687	−0.097
(rest * early-post-move)	0.31	0.151	0.015	0.606
(social human * early-post-move)	−1.223	0.151	−1.518	−0.927
(social negative * early-post-move)	−0.19	0.151	−0.485	0.106
(social positive * early-post-move)	1.794	0.151	1.498	2.089
(vigilance * early-post-move)	0.532	0.151	0.236	0.827
(autogroom * post-move)	0.098	0.096	−0.089	0.285
(feed * post-move)	−1.042	0.096	−1.229	−0.855
(forage * post-move)	0.046	0.096	−0.141	0.234
(locomotion * post-move)	−0.253	0.096	−0.44	−0.066
(object manipulation * post-move)	0.785	0.096	0.598	0.972
(play * post-move)	−0.52	0.096	−0.707	−0.333
(rest * post-move)	0.262	0.096	0.075	0.449
(social human * post-move)	−2.444	0.096	−2.631	−2.257
(social negative * post-move)	0.04	0.096	−0.147	0.228
(social positive * post-move)	2.478	0.096	2.290	2.665
(vigilance * post-move)	0.065	0.096	−0.122	0.252
Random Effects:				
Residual variance	6.24			
(individual) variance	0			
(individual) standard deviation	0			
(observation session) variance	0			
(observation session) standard deviation	0			
N (individual)	6			
N (observation session)	190			
Observations	2280			
Marginal R^2^/Conditional R^2^	0.341/NA			

**Table A6:** Model 2 summary output: Crested macaque activity budget.

Term	Estimate	SE	Lower CI	Upper CI
(Intercept)	−2.717	0.051	−2.818	−2.616
Behaviour:				
(autogroom)	1.729	0.066	1.599	1.859
(feed)	2.239	0.066	2.109	2.369
(forage)	3.069	0.066	2.939	3.199
(locomotion)	4.392	0.066	4.262	4.523
(play)	1.298	0.066	1.168	1.428
(rest)	6.576	0.066	6.446	6.706
(social negative)	0.158	0.066	0.028	0.289
(social positive)	4.989	0.066	4.859	5.119
Enclosure-move condition:				
(early-post-move)	−0.03	0.1	−0.225	0.166
(post-move)	0.07	0.08	−0.087	0.228
Sex:				
(female)	0	0.028	−0.054	0.054
Age (days)	0	0.012	−0.023	0.023
Behaviour * Enclosure-move condition:				
(autogroom * early-post-move)	0.159	0.141	−0.118	0.436
(feed * early-post-move)	−0.821	0.141	−1.098	−0.544
(forage * early-post-move)	0.083	0.141	−0.194	0.359
(locomotion * early-post-move)	−0.092	0.141	−0.369	0.185
(play * early-post-move)	0.192	0.141	−0.085	0.469
(rest * early-post-move)	−0.568	0.141	−0.845	−0.291
(social negative * early-post-move)	−0.158	0.141	−0.435	0.118
(social positive * early-post-move)	1.471	0.141	1.194	1.747
(autogroom * post-move)	1.365	0.113	1.143	1.587
(feed * post-move)	−0.952	0.113	−1.175	−0.730
(forage * post-move)	−0.841	0.113	−1.063	−0.618
(locomotion * post-move)	−0.773	0.113	−0.995	−0.550
(play * post-move)	−0.346	0.113	−0.568	−0.123
(rest * post-move)	0.11	0.113	−0.112	0.332
(social negative * post-move)	−0.116	0.113	−0.338	0.106
(social positive * post-move)	0.919	0.113	0.697	1.141
Random Effects:				
Residual variance	8.637			
(individual) variance	0			
(individual) standard deviation	0			
(observation session) variance	0			
(observation session) standard deviation	0			
N (individual)	13			
N (observation session)	645			
Observations	5805			
Marginal R^2^/Conditional R^2^	0.356/NA			

**Table A7:** Model 3 summary output: Malayan sun bear activity budget.

Term	Estimate	SE	Lower CI	Upper CI
(Intercept)	−0.419	0.048	−0.513	−0.324
Behaviour:				
(explore)	3.121	0.045	3.033	3.209
(feed)	−0.198	0.045	−0.285	−0.110
(forage)	1.292	0.045	1.204	1.38
(locomotion)	4.278	0.045	4.191	4.366
(object manipulation)	−1.6	0.045	−1.688	−1.512
(rest)	2.359	0.045	2.271	2.446
(social human)	−1.797	0.045	−1.885	−1.709
(social negative)	−1.748	0.045	−1.836	−1.660
(social positive)	−1.519	0.045	−1.607	−1.432
Enclosure-move condition:				
(early-post-move)	−1.089	0.077	−1.24	−0.937
Sex				
(female)	0	0.079	−0.156	0.156
Age (days)	0	0.041	−0.081	0.081
Behaviour * Enclosure-move condition:				
(explore * early-post-move)	1.733	0.103	1.531	1.934
(feed * early-post-move)	0.798	0.103	0.597	0.999
(forage * early-post-move)	0.623	0.103	0.422	0.824
(locomotion)* early-post-move)	0.83	0.103	0.629	1.032
(object manipulation * early-post-move)	0.935	0.103	0.733	1.136
(rest * early-post-move)	3.207	0.103	3.006	3.409
(social human * early-post-move)	0.942	0.103	0.741	1.143
(social negative * early-post-move)	0.76	0.103	0.559	0.961
(social positive * early-post-move)	1.06	0.103	0.858	1.261
Random Effects				
Residual variance	5.964			
(observation session) variance	0			
(observation session) standard deviation	0			
N (observation session)	279			
Observations	2790			
Marginal R^2^/Conditional R^2^	0.444/NA			

**Table A8:** Model 4 summary output: Malayan tapir activity budget.

Term	Estimate	SE	Lower CI	Upper CI
(Intercept)	−1.135	0.017	−1.168	−1.103
Behaviour:				
(feed)	3.667	0.022	3.625	3.71
(forage)	1.682	0.022	1.639	1.725
(locomotion)	2.293	0.022	2.250	2.336
(play)	−1.375	0.022	−1.418	−1.332
(rest)	5.143	0.022	5.100	5.185
(social negative)	−1.531	0.022	−1.574	−1.488
(social positive)	−0.796	0.022	−0.839	−0.753
Enclosure-move condition:				
(early-post-move)	1.475	0.071	1.336	1.614
(post-move)	−0.59	0.023	−0.636	−0.544
Sex:				
(female)	0	0.009	−0.019	0.019
Age (days)	0	0.004	−0.009	0.009
Behaviour * Enclosure-move condition:				
(feed * early-post-move)	−3.667	0.1	−3.863	−3.471
(forage * early-post-move)	−1.184	0.1	−1.38	−0.988
(locomotion * early-post-move)	−0.764	0.1	−0.96	−0.568
(play * early-post-move)	−0.397	0.1	−0.593	−0.201
(rest * early-post-move)	−2.104	0.1	−2.3	−1.908
(social negative * early-post-move)	−1.474	0.1	−1.67	−1.278
(social positive * early-post-move)	−2.209	0.1	−2.405	−2.013
(feed * post-move)	0.282	0.033	0.218	0.347
(forage * post-move)	0.646	0.033	0.581	0.711
(locomotion * post-move)	2.615	0.033	2.550	2.68
(play * post-move)	0.677	0.033	0.612	0.741
(rest * post-move)	−1.187	0.033	−1.252	−1.123
(social negative * post-move)	0.477	0.033	0.412	0.541
(social positive * post-move)	1.213	0.033	1.148	1.277
Random Effects:				
Residual variance	1.743			
(observation session) variance	0			
(observation session) standard deviation	0			
N (observation session)	96			
Observations	768			
Marginal R^2^/Conditional R^2^	0.744/NA			

**Table A9:** Raw mean proportions (with standard errors) of time spent in combined behavioural categories (expressed as a percentage of the total activity budget) under the pre-, early-post- and post-enclosure-move conditions for each species studied. ‘n’ refers to the number of sampling points for each data collection condition.

Species	Behaviour	Pre (% (SE) n)		Early Post (% (SE) n)		Post (% (SE) n)	
Sumatran orangutan	Abnormal	0 (0.0)	81	0.1 (0.1)	23	0 (0)	86
	Autogroom	4.8 (0.8)	81	6.7 (1.6)	23	3.4 (0.5)	86
	Explore	0 (0.0)	81	0 (0)	23	0 (0)	86
	Feed	11 (1.8)	81	11.2 (2.4)	23	0 (0)	86
	Forage	4.8 (0.9)	81	0.8 (0.6)	23	7.5 (1.2)	86
	Interaction with humans	10.2 (2.0)	81	1.6 (0.6)	23	4.1 (0.8)	86
	Locomotion	12.5 (1.1)	81	14.8 (1.7)	23	0.1 (0.1)	86
	Negative social	0.2 (0.1)	81	0 (0)	23	11 (1.2)	86
	Object manipulation	4.9 (1.0)	81	2.7 (0.9)	23	0 (0)	86
	Play	14.8 (2.3)	81	10.6 (3.7)	23	8.7 (1.5)	86
	Positive social	7.5 (1.3)	81	11.5 (2.9)	23	11.3 (2.1)	86
	Rest	6.8 (1.7)	81	4.7 (1.7)	23	19.6 (2.3)	86
	Vigilance	22.3 (2.6)	81	34.3 (5.7)	23	12.8 (2.4)	86
Crested macaque	Abnormal	0 (0)	358	0 (0)	23	21.4 (2.4)	86
	Autogroom	4.8 (0.7)	358	5.7 (1.3)	101	0 (0)	186
	Explore	0 (0)	358	0 (0)	101	10.5 (1.3)	186
	Feed	9.2 (1.0)	358	4 (1.2)	101	0 (0)	186
	Forage	11.0 (1.0)	358	12.3 (2.0)	101	4.3 (1.0)	186
	Interaction with humans	0 (0)	358	0 (0)	101	8.1 (1.3)	186
	Locomotion	11.4 (0.7)	358	10.4 (1.3)	101	0 (0)	186
	Negative social	0.3 (0.1)	358	0 (0)	101	8.5 (0.8)	186
	Object manipulation	0 (0)	358	0 (0)	101	0.1 (0.1)	186
	Play	3.7 (0.6)	358	3.7 (0.9)	101	0 (0)	186
	Positive social	28.8 (1.8)	358	41.4 (3.7)	101	2.1 (0.5)	186
	Rest	30.8 (1.4)	358	22.6 (2.0)	101	36.7 (2.5)	186
	Vigilance	0 (0)	358	0 (0)	101	29.7 (1.8)	186
Malayan sun bear	Abnormal	9.6 (1.3)	237	4.1 (2)	101	0 (0)	186
	Autogroom	0 (0)	237	0 (0)	-	-	-
	Explore	17.5 (1.2)	237	24.1 (3.1)	-	-	-
	Feed	3.7 (0.6)	237	3.7 (1.7)	-	-	-
	Forage	13.3 (1.4)	237	9.2 (2.2)	-	-	-
	Interaction with humans	0.2 (0.2)	237	0.2 (0.2)	-	-	-
	Locomotion	26.2 (1.5)	237	19.9 (2.2)	-	-	-
	Negative social	0.1 (0.1)	237	0 (0)	-	-	-
	Object manipulation	1.2 (0.5)	237	1.2 (0.9)	-	-	-
	Play	0 (0)	237	0 (0)	-	-	-
	Positive social	1 (0.4)	237	0.5 (0.2)	-	-	-
	Rest	27.1 (2.3)	237	37.2 (5)	-	-	-
	Vigilance	0 (0)	237	0 (0)	-	-	-
Tapir	Abnormal	0 (0)	51	0 (0)	-	-	-
	Autogroom	0 (0)	51	0 (0)	3	0 (0)	42
	Explore	0.8 (0.2)	51	3.9 (2.0)	3	0 (0)	42
	Feed	25.7 (3.3)	51	3.9 (2.0)	3	2.3 (1.2)	42
	Forage	3.8 (0.7)	51	9.2 (5.8)	3	28.2 (3.3)	42
	Interaction with humans	0 (0)	51	0 (0)	3	6.7 (1.0)	42
	Locomotion	6.2 (0.9)	51	39.1 (19.8)	3	0 (0.0)	42
	Negative social	0 (0)	51	0 (0)	3	37.9 (3.0)	42
	Object manipulation	0 (0)	51	0 (0)	3	0 (0)	42
	Play	0.1 (0.1)	51	0.9 (0.9)	3	0 (0)	42
	Positive social	0.7 (0.4)	51	0 (0)	3	0.2 (0.1)	42
	Rest	62.7 (3.9)	51	42.9 (28.6)	3	1.4 (0.3)	42
	Vigilance	0 (0)	51	0 (0)	3	23.3 (2.9)	42

**Table A10:** Model 5 summary output: Effect of enclosure move on Sumatran orangutan visibility.

Term	Estimate	SE	Lower CI	Upper CI
(Intercept)	0.162	0.031	0.112	0.235
Enclosure-move condition:				
(early-post-move)	0.725	0.251	0.368	1.431
(post-move)	1.164	0.228	0.793	1.708
Sex:				
(female)	0.919	0.191	0.611	1.382
Age (days)	0.958	0.089	0.798	1.149
Random Effects:				
(individual) variance	0			
(individual) standard deviation	0			
N (individual)	6			
Observations	63			
Marginal R^2^/Conditional R^2^	0.055/NA			

**Table A11:** Model 6 summary output: Effect of enclosure move on crested macaque visibility.

Term	Estimate	SE	Lower CI	Upper CI
(Intercept)	0.275	0.151	0.094	0.805
Enclosure-move condition:				
(early-post-move)	1.024	0.552	0.356	2.948
(post-move)	1.209	0.64	0.428	3.414
Sex:				
(female)	1.213	0.206	0.870	1.691
Age (days)	0.936	0.072	0.806	1.088
Random Effects				
(individual) variance	0			
(individual) standard deviation	0			
N (individual)	13			
Observations	98			
Marginal R^2^/Conditional R^2^	0.088/NA			

**Table A12:** Model 7 summary output: Effect of enclosure move on Malayan sun bear visibility.

Term	Estimate	SE	Lower CI	Upper CI
(Intercept)	0.713	0.232	0.377	1.348
Enclosure-move condition:				
(early-post-move)	0.524	0.162	0.285	0.962
Sex:				
(female)	2.789	1.886	0.741	10.5
Age (days)	1.414	0.494	0.712	2.805
Observations	266			

**Table A13:** Model 8 summary output: Effect of enclosure move on Malayan tapir visibility.

Term	Estimate	SE	Lower CI	Upper CI
(Intercept)	0.439	0.194	0.184	1.044
Enclosure-move condition:				
(early-post-move)	16.194	9.514	5.120	51.217
(post-move)	6.237	2.853	2.545	15.289
Sex:				
(female)	0.717	0.235	0.378	1.362
Age (days)	0.936	0.15	0.684	1.282
Observations	63			
